# Editorial: Pioneers & pathfinders: 10 years of frontiers in medicine

**DOI:** 10.3389/fmed.2025.1736691

**Published:** 2025-11-18

**Authors:** M. Goldman, A. Chen, J. Bloomfield, V. Bunik, C. T. Chao, J. E. Fonseca, E. Gavriilaki, R. Gniadecki, A. M. Kaynar, A. Lanas, B. Lima, A. G. Mainous, L. V. Monrouxe, G. Treglia

**Affiliations:** 1 Institute for Interdisciplinary Innovation in Healthcare, Université libre de Bruxelles, Brussels, Belgium; 2 AP Chen Consultant, Potomac, MD, United States; 3 The University of Sydney, Sydney, NSW, Australia; 4 Belozersky Institute of Physicochemical Biology and Faculty of Bioengineering and Bioinformatics, Lomonosov Moscow State University, Moscow, Russia; 5 Department of Internal Medicine, National Taiwan University Hospital, Taipei, Taiwan; 6 Faculdade de Medicina, Universidade de Lisboa and ULS Santa Maria, Lisbon, Portugal; 7 2nd Propedeutic Department of Internal Medicine, Hippocration Hospital, Aristotle University of Thessaloniki, Thessaloniki, Greece; 8 University of Alberta, Edmonton, AB, Canada; 9 University of Pittsburgh School of Medicine, Pittsburgh, PA, United States; 10 Aragón Health Research Institute (IIS Aragón), Zaragoza, Spain; 11 Centro de Investigacion Biomédica en Red de enfermedades hepaticas y digestivas (CIBERehd), Zaragoza, Spain; 12 University of Florida, Gainesville, FL, United States; 13 Clinic of Nuclear Medicine, Ente Ospedaliero Cantonale, Bellinzona, Switzerland; 14 Faculty of Biology and Medicine, University of Lausanne, Lausanne, Switzerland; 15 Faculty of Biomedical Sciences, Università della Svizzera italiana, Lugano, Switzerland

**Keywords:** artificial intelligence, precision medicine, pharmaceuticals, innovation, public trust

During the last decade, unprecedented advances were made in the field of medical science. The concept of precision medicine that revolutionized the treatment of cancer disorders progressively extended to other medical areas and millions of lives were saved by mRNA vaccines during the COVID-19 pandemic. Furthermore, disruptive technologies based on genetic materials offered new therapeutic perspectives for several rare diseases. There are many other examples of the spectacular evolution of healthcare in recent years. Several challenges are still ahead of us not only from a purely scientific perspective, but also to adapt regulatory policies and to ensure that innovative solutions are accessible to every patient.

As Frontiers in Medicine celebrates its 10th anniversary as a journal in the top 25% of its category, we invited authors to submit papers reporting what they considered as meaningful advances, worth publishing in different sections of the journal as part of this Research Topic. Based on the contributions received and the input of our section editors, we mention here below key developments in different medical disciplines—emphasizing the growing impact of artificial intelligence (AI).

Indeed, the paper by Lin et al. highlighted the explosive growth of artificial intelligence (AI) in healthcare, documenting over 1,800 publications from 97 countries between 2019 and 2023 in their bibliometric analysis of AI in medicine. Their study identified key progress areas, emerging fields, and leading contributors—including prominent countries, institutions, and researchers—providing valuable insights into current collaborative frameworks and potential future research directions (Lin et al.). Among the many domains where AI is making an impact, precision oncology exemplifies its transformative potential, enabling more personalized cancer care through enhanced diagnostic accuracy, Hashem and Sultan have emphasized its growing impact in pediatric oncology, where AI-driven innovations hold promise for improving diagnosis and tailoring therapies for young patients. Despite these promising advances, the field remains in its infancy, and significant implementation challenges persist. While the success of AI in precision medicine underscores its ability to address complex medical problems, access to advanced tools remains confined to well-resourced healthcare systems. Moving forward, sustained progress will depend on the establishment of rigorous methodological standards, robust ethical frameworks, and the integration of real-world data with the goal of benefiting all global population. As part of this Research Topic, the current and anticipated contributions of AI are also discussed in dermatology (Gniadecki), gastroenterology (Aso et al.), and intensive care/anesthesiology (Kaynar), nephrology (Yoo and Chao; Hu et al.) and rheumatology (Vieira-Sousa et al.; Serban et al.). Clearly, regulatory science and public health (Schweizer-Schubert et al.) will also benefit from AI developments. In this new era, it will be essential to maintain public trust in the recommendations made by experts, taking into consideration that the opinions expressed might be conflicting and influenced by political considerations (Mainous et al.).

Furthermore, the tremendous potential of analytical techniques for deciphering genotype-phenotype relationship has been emphasized by Bunik. She underlines that the field requires development of public databases on genetic variety and associated disease diagnostics, as well as specific programs in medical education.

Several other themes are covered in this Research Topic, including new applications of radiopharmaceuticals in oncology and autoimmune diseases (Lepareur; Frank et al.) as well as new targeted therapeutic modalities in hematology (Gavriilaki; Gavriilaki et al.). We also received an important contribution on the impact of education of healthcare professions with a focus on emotional intelligence (Maity).

We warmly hope that the value of this series of articles will be recognized and incentivize new submissions to our journal which is now established as a flagship among open access medical publications.

